# A Readily Soluble Hypnotic of the Veronal Group

**Published:** 1908-10

**Authors:** 


### A Readily Soluble Hypnotic of the Veronal Group.

Ernst Steinitz (*Therapie der Gegenwart,* July, 1908,) says: the ideal hypnotic must be readily soluble and, therefore, rapidly absorbed, so that the soporific effect may be prompt and certain and that there may be no undesired prolongation of it. Diethyl-barbituric acid, one of the best hypnotics known, has the disadvantage of very slight solubility (1:145).

Steinitz experimented clinically with the monosodium salt of diethyl-barbituric acid, placed at his disposal by Schering. It is a crystalline powder 20 per cent. soluble in water; by heating a 30 per cent. solution, permanent in the cold, can be prepared. He used it by mouth, per rectum and subcutaneously.

By mouth the salt is always given dissolved in water, when it has no unpleasant taste. Its action was quicker and more certain than that of diethyl-barbituric acid; occasionally the salt had good effect where the base had been ineffective. A cumulative toxic action was absent, though in some cases 7½ grains were given daily for considerable periods.

In most cases the superiority of the new salt is doubtless due to its being administered thoroughly dissolved. It is best taken on an empty and consequently acid-free stomach—on retiring—when it passes unchanged into the intestines and is there quickly absorbed. In the ratio in which absorption and therefore excretion are hastened, the salt naturally diminishes the danger of cumulative toxic actions.

Per rectum the action of the drug was quicker and almost always more intensive. In some cases the effect was excellent when the same dose by mouth had no effect at all. Anesthetic properties were also observed at times. In several cases of cardiac and bronchial asthma, with night attacks, the greatest possible relief was effected by the rectal injections. Seven and one-half grains were dissolved in about a dram of water and injected with a small rectal syringe.

Subcutaneous injections are intensive rather than rapid in effect. Seventy-five minims of a 10 per cent. solution are used; this hardly irritates and is apparently more quickly absorbed than more concentrated solutions. Abscesses never occurred, though some patients were morphinists who in other places were sown with injection abscesses. In threatened delirium tremens the salt has about replaced chloral by subcutaneous injection. The injection of the salt seemed especially suitable, too, for patients under antimorphine treatment; here its quietening effects were marked.

Steinitz concludes: "On account of its extreme water solubility, the salt can be conveniently administered in a well dissolved and most finely subdivided state. The absorption proceeds more quickly when it is administered on an acid-free stomach and when given per rectum, because the drug remains in its readily soluble form.

Rectal administration can be recommended when the stomach is to be spared and especially for obstinate insomnias.

Subcutaneous injection we recommend at present only in special cases, where the patients refuse oral medication, for morphine habit, and as a last resort in severest insomnia."

---

SAMUEL WYLLIS BANDLER, New York, in his *Medical Gynecology* (W. B. Saunders Co., 1908,) has the following passage: Acute female gonorrheal urethritis: Of the internal antigonorrheic remedies, arhovin is said to be free from the untoward effects of the balsams. Arhovin internally is indicated in inflammation of the urethra associated with pain on urination and cystitis. In the chronic form it sometimes gives great relief. Arhovin causes acidity in alkaline urine, even in one undergoing ammoniacal fermentation. It is sedative and anesthetic to mucosæ and does not annoy the stomach. Six to twelve capsules, each containing 4 grains, are to be taken daily.

---

ONE of the most important criteria of a well-fitting pessary is that the finger can pass fairly easily between its sides and the vaginal wall.—*International Journal Surgery.*

